# *Limoniastrum guyonianum* aqueous gall extract induces apoptosis in human cervical cancer cells involving p16^INK4A^ re-expression related to UHRF1 and DNMT1 down-regulation

**DOI:** 10.1186/1756-9966-32-30

**Published:** 2013-05-20

**Authors:** Mounira Krifa, Mahmoud Alhosin, Christian D Muller, Jean-Pierre Gies, Leila Chekir-Ghedira, Kamel Ghedira, Yves Mély, Christian Bronner, Marc Mousli

**Affiliations:** 1 UMR CNRS 7213, Laboratoire de Biophotonique et Pharmacologie, Faculté de Pharmacie, Université de Strasbourg, 74 Route du Rhin, CS 60024, Illkirch, Cedex F-67401, France; 2 Unité de Pharmacognosie/Biologie Moléculaire 99/UR/07-03. Faculté de Pharmacie de Monastir, Rue Avicenne 5000, Monastir, Tunisie; 3 UMR CNRS 7200, Laboratoire d’Innovation Thérapeutique, Université de Strasbourg, Faculté de Pharmacie, 74 route du Rhin, 67401 Illkirch, France; 4 Institut de Génétique et de Biologie Moléculaire et Cellulaire, CNRS/INSERM, Parc d’innovation, 1 rue Laurent Fries, Illkirch, Cedex 67404, France

**Keywords:** Apoptosis, Epigenetic modifications, Gall extract, Tumor suppressor genes, UHRF1

## Abstract

Several reports have described the potential effects of natural compounds as anti-cancer agents *in vitro* as well as *in vivo*. The aim of this study was to evaluate the anti-cancer effect of *Limoniastrum guyonianum* aqueous gall extract (G extract) and luteolin in the human cervical cancer HeLa cell line, and, if so, to clarify the underlying mechanism. Our results show that G extract and luteolin inhibited cell proliferation and induced G2/M cell cycle arrest in a concentration and time-dependent manner. Both natural products induced programmed cell death as confirmed by the presence of hypodiploid G0/G1 cells. These effects are associated with an up-regulation of the expression of the tumor suppressor gene *p16*^*INK4A*^ and a down-regulation of the expression of the anti-apoptotic actor UHRF1 and its main partner DNMT1. Moreover, G extract- and luteolin-induced UHRF1 and DNMT1 down-regulation is accompanied with a global DNA hypomethylation in HeLa cell line. Altogether our results show that G extract mediates its growth inhibitory effects on human cervical cancer HeLa cell line likely via the activation of a p16^INK4A^ -dependent cell cycle checkpoint signalling pathway orchestrated by UHRF1 and DNMT1 down-regulation.

## Introduction

Natural products derived from plants have received extensive attention as potential anti-cancer agents over few decades. Most of current anti-cancer drugs such as camptothecin, vincristine, taxol, etoposide and paclitaxel are plant-derived compounds [1,2]. These bioactive phytochemicals are known to exert their anti-cancer activity through different mechanisms, including altered carcinogen metabolism, induction of DNA repair systems, immune activation, suppression of cell cycle progression and induction of apoptosis. Several studies have shown that natural products rich in polyphenols have strong chemopreventive and chemotherapeutic properties in different types of cancer cells [3,4]. Flavonoids, polyphenolic compounds found in plant-derived dietary components, exhibit multiple biological activities, including anticarcinogenic activity. Luteolin, one of the most effective flavonoids, can delay or block the development of cancer cells *in vitro* and *in vivo* via inhibition of tumor cell proliferation, induction of cell cycle arrest and apoptosis by inhibiting enzymes involved in cell activation such as phosphodiesterases kinases and DNA topoisomerases [5].

Methylation of CpG islands is an important component of the epigenetic code and a number of genes become abnormally methylated during tumorigenesis. A hypermethylation of the tumor suppressor gene *p16*^*INK4A*^ at its CpG-rich promoter regions and subsequent inactivation of the *p16*^*INK4A*^ gene have been reported in several haematological and solid cancers [6,7]. This hypermethylation targets the expression of specific genes involved in the DNA damage response including, the retinoblastoma protein (pRB) [8]. More recently, many studies have reported that UHRF1 serves as a fidelity factor for the maintenance of the DNA methylation pattern throughout cell duplication [9,10]. The Set and Ring Associated domain (SRA domain) of UHRF1 has the unique feature to recognize a particular state of DNA, *i.e.,* hemi-methylated. The SRA–DNA interaction may serve as an anchor to keep UHRF1 at a hemi-methylated CpG site where it recruits the DNMT1 for DNA methylation maintenance [9,11]. Thus, UHRF1 plays a fundamental role in the inheritance of the DNA epigenetic marks from the mother cell to the daughter cells. It also appears that preventing the transmission of these marks via knock-down of UHRF1 leads to an activation of pro-apoptotic pathways [9,12-16]. In agreement with this hypothesis, UHRF1 down-regulation has been shown to inhibit cell growth and induces apoptosis of colorectal cancer through p16^INK4A^ up-regulation [17]. Some bioactive plants components have been shown to have cancer inhibition activities by reducing DNA hypermethylation of key cancer-causing genes through their DNA methyltransferase (DNMT) inhibition properties [18]. In this context, recently we found that the epigallocatechin-3-gallate (EGCG), a natural anti-cancer drug induces G1 cell arrest and apoptosis in Jurkat cells by down-regulating UHRF1 and DNMT1 expression, with subsequent up-regulation of *p16*^*INK4A*^ gene [19].

*L. guyonianum* has been used in traditional medicines to treat gastric infections. It has also been employed as an anti-bacterial drug in the treatment of bronchitis [20]. *L. feei* has been similarly used in the treatment of bronchitis and stomach infections [21]. Previous investigations revealed that methanol extract from *L. feei* leaves contained potential anti-fungal constituents that could be employed against *Candida albicans* and anti-bacterial constituents useful against *E. coli*[22]. More recently, our laboratory demonstrated that *L. guyonianum* aqueous gall extract was able to induce splenocyte proliferation and to stimulate macrophage activation [23].

Chemical investigation of *Limoniastrum genus* has been reported in literature. Indeed, bioguided fractionation of leaves extract from *Limoniastrum feei* led to the isolation of several polyphenolic constituents such as Gallic acid, Epigallocatechin gallate, Quercetin and Myricetin [24]. A subsequent article noted that ethyl acetate extract of *L. guyonianum* contained gallocatechin, epigallocatechin, and epigallocatechin-3-O-gallate [25]. Several groups have reported that epigallocatechin gallate exhibited antitumor effects that were discovered from various cancer cell lines, animal models and clinical studies [26]. For example, *in vivo* studies showed that epigallocatechin gallate administration decreased H1299 xenograft tumor growth [27]. Furthermore, myricetin treatment significantly inhibited the tumor growth on T24 bladder cancer xenografts model [28]. In the same way, it was demonstrated that gallic acid plays a critical role as an anticancer agent *in vivo* by decreasing MNNG/HOS xenograft tumor growth in Balb/C mice [29].

The aim of this study was to determine, in HeLa cervical cancer cell line, whether *Limoniastrum guyonianum* aqueous gall extract and luteolin, one of the most common flavonoids, could target UHRF1 and DNMT1 expression with subsequent cell cycle arrest and apoptosis via up-regulation of *p16*^*INK4A*^ gene. Our results show that G extract and luteolin cause G2/M cell cycle arrest and trigger apoptosis likely through the inhibition of UHRF1/DNMT1 tandem expression, followed by an up-regulation of *p16*^*INK4A*^.

## Materials and methods

### Materials

*Limoniastrum guyonianum* samples were collected from El Hamâ at Gabbes (a region situated in southern Tunisia). Dr. Fethia Skhiri (Department of Botany, Higher Institute of Biotechnology, University of Monastir) performed sample identification and verification according to the Tunisian Guide on Flora [30]. A voucher specimen (#L.g-10.09) was preserved for future reference. Luteolin (> 90% of purity) was purchased from Extrasynthese (Genay, France). 3-(4,5-dimethylthiazol-2-yl)-2,5-diphenyltetrazoliumbromide (MTT) was from Euromedex (Mundolsheim, France), propidium iodide (PI), Tris Buffered Saline with tween 20 (TBST) and dimethylsulfoxide (DMSO) from Sigma-Aldrich (St. Quentin Fallavier, France). Dulbecco’s Modified Eagle’s Medium (DMEM), fetal calf serum (FCS), trypsin and L-glutamine were purchased from Invitrogen Life Technologies (Cergy Pontoise, France). Folin-Ciocalteu phenol reagent was obtained from BDH laboratory (Poole, England). Sodium carbonate (Na_2_CO_3_) was purchased from Acros Organics (Geel, Belgium). Nitrite sodium (NaNO_2_) and aluminum chloride (AlCl_3_) were procured from Aldrich (Steinheim, Germany).

### Preparation of plant extract

The collected gall samples were shade-dried, powdered, and then stored in a tightly closed container for further use. When needed, powdered gall (100 g) was extracted in boiling water (1 L) for 15–20 min and after filtration, the aqueous extract was frozen and then lyophilized and kept at 4°C. The total aqueous extract concentrate yield (per gram dried plant material) was determined using the formula: 100 x weight (g) of dried extract/dry-weight (g) of plant material. The actual percentage yield in this study was 17.8%. From this material, extract solutions containing different concentrations from 100 to 300 μg/ml were then prepared for use in the evaluation of their cytotoxic and pro-apoptotic effects on HeLa cells. The polyphenol content of *L. guyonianum* gall aqueous extract was quantified by the Folin-Ciocalteau method [31,32] and was expressed as gallic acid equivalent. Aliquots of test sample (100 μl) were mixed with 2.0 ml of 2% Na_2_CO_3_ and incubated at room temperature for 2 min. After the addition of 100 μl of 50% Folin-Ciocalteau phenol reagent, the reaction tube was incubated for 30 min at room temperature, and finally absorbance was read at 720 nm. A known volume of the extract was placed in a 10 ml volumetric flask to estimate flavonoid content [33]. After addition of 75 μl of NaNO_2_ (5%), 150 μl of freshly prepared AlCl_3_ (10%), and 500 μl of NaOH (1 N), the volume was adjusted with distilled water until 2.5 ml. After 5 min incubation, the total absorbance was measured at 510 nm. Quercetin was used as a standard for constructing a calibration curve.

The method described by [34] was used for the determination of tannin content of samples. Extraction of tannins was achieved by dissolving 5 g of sample in 50 ml of distilled water in a conical flask, allowing the mixture to stand for 30 min with shaking the flask at 10 min intervals, and then centrifuging at 5000 *g* to obtain a supernatant (tannin extract). The extract was diluted to 100 ml in a standard flask using distilled water. Five milliliters of the diluted extract and 5 ml of standard tannic acid (0.1 g/L) were measured into different 50 ml volumetric flasks. One milliliter of Folin-Denis reagent was added to each flask followed by 2.5 ml of saturated sodium carbonate solution. The solutions were made up to the 50 ml mark with distilled water and incubated at room temperature (20–30°C) for 90 min. The absorption of these solutions was measured against the reagent blank (containing 5 ml distilled water in the place of the extract or the standard tannic acid solution) at 760 nm wavelength. Tannin content was calculated in triplicates as: sample reading/standard reading × 20 [35].

### Cell culture

The human cervical cancer cell line HeLa was obtained from the American Type Culture Collection (Rockville, Maryland, USA) and maintained in a humidified incubator with 5% CO2 at 37°C, and grown in DMEM (Dulbecco’s Modified Eagle’s Medium). The medium was supplemented with 10% (v/v) fetal calf serum (FCS, Biowhitaker, Lonza, Belgium), 2 mM glutamine, 100 U/ml penicillin and 50 mg/ml streptomycin (Sigma St. Louis, MO).

### Cell proliferation and apoptosis assays

Cells were seeded in 96-well cell culture plates at a density of 0.5 × 10^4^ cells/well, grown for 24 hours and exposed to different concentrations of G extract or luteolin for 24 hours. Cell proliferation rate was then assessed by colorimetric assay using the CellTiter 961 Aqueous One Solution Cell Proliferation Assay (MTT), following the manufacturer’s recommendations. Early and late apoptosis were monitored by flow cytometry (Guava PCA-96 Merck/Millipore, Molsheim, France). To discriminate between negative and positive events in the analysis, a non-stained control sample from each culture condition always accompanied acquisition of the stained cells to define their cut off. Gates were drawn around the appropriate cell populations using a forward scatter (FSC) versus side scatter (SSC) acquisition dot plot. Late apoptotic cells are double labelled by Annexin V and 7-AAD (Guava Nexin Reagent kit Merck/Millipore). Cytometers performances are checked weekly using the Guava easyCheck Kit 4500–0025 (Merck/Millipore/Guava Hayward, CA, USA).

### Cell cycle analysis

Cells were seeded in 96-well cell culture plates at a density of 0.5 × 10^4^ cells/well and grown for 24 hours, then exposed to different concentrations of G extract or luteolin for 24 hours. Cells were then washed with phosphate-buffered saline (PBS), trypsin detached then fixed with paraformaldehyde (1%). After 30 min incubation at room temperature, 5 μl of propidium iodide was added in each well (1 μg/ml). Cellular DNA content was assessed by capillary cytometry (Guava EasyCyte 96 Plus). Data were analyzed on the Guava CytoSoft™ Express Pro software (Merck/Milli pore/Guava Tech). CytoSoft Express Pro was used to identify the three cell cycle phases and calculate relevant statistics, including population percentages (subG1, G0/G1, S and G2/M phases).

### Quantification of DNA methylation

HeLa cells were treated with G extract (200 μg/ml) or luteolin (25 μM) for 48 hours. DNA was purified using QIAamp® DNA Kit. The content of methylated DNA was determined using 200 ng of DNA from untreated cells, treated cells with G extract or luteolin, as described by the manufacturer; Sigma’s Imprint® Methylated DNA Quantification Kit.

### Western blot analysis

HeLa cells (6 × 10^5^) were seeded into 6-well cell culture plates and grown for 24 hours. Cells were treated with different concentrations of G extract or luteolin for 24 and 48 hours. The cells were then harvested, centrifuged to discard the DMEM medium, washed with cold PBS (phosphate buffered saline), resuspended in RIPA buffer (25 mM Tris, pH 7.6, 150 mM NaCl, 1% NP-40, 1% sodium deoxycholate and 0.1% SDS; Sigma–Aldrich, USA) containing protease inhibitors. Equal amounts of total protein were separated on 10–12% polyacrylamide gel and electrophoretically transferred to a nitrocellulose membrane. After blocking with 5% non-fat dry milk or 3% BSA (Bovine Serum Albumin) and tween 20 in PBS, the nitrocellulose membranes were incubated with either a mouse monoclonal anti-UHRF1 antibody (Proteogenix, Oberhausbergen, France), a mouse monoclonal anti-DNMT1 (clone 60B1220.1, Proteogenix), and a rabbit polyclonal anti-p16^INK4A^ antibody (DeltaBiolabs, Gilroy, CA) according to the manufacturer’s instructions (4°C, overnight). Membranes were thereafter incubated with the appropriate horseradish peroxidase-conjugated secondary antibody (diluted to 1:10,000 for anti-mouse antibodies and 2: 10,000 for anti-rabbit antibody) at room temperature for 45 minutes. The membranes were then washed with TPBS five times. Signals were detected by chemiluminescence using the ECL Plus detection system (Amersham, GE Healthcare UK Limited).

### Statistical analysis

Data were analyzed with student’s *t*-test and presented as mean value ± *S.E.M* of three independent measurements in separate experiments.

## Results

### Aqueous gall extract content

Aqueous gall extract from *L. guyonianum* was the subject of a chemical study with the aim of having a global idea in their composition. The metabolites contents of the tested extract are presented in Table 1. Quantitative phytochemical analysis showed that the extract contained an important quantity of flavonoids, polyphenols, and tannins. In fact, 1 mg of G extract was equivalent to 85 μg of gallic acid and 460 μg of quercetin. The higher content of tannins was also recorded in the extract with 77.5 mg/100 g.

**Table 1:** **Phytochemical composition of aqueous gall (G) extract from *L.guyonianum***

**Metabolites**	**Extract content (μg)**
**Flavonoids (Quercetin equivalent)**	**460 ± 14**
**Polyphenols (Gallic acid equivalent)**	**85 ± 6**
**Tannis (mg/100g tannic acid)**	**77 ± 5**

Values are means ± *S.E.M.* of three independent experiments.

### Aqueous gall extract and luteolin induce UHRF1 and DNMT1 down-regulation and p16^INK4A^ up-regulation associated with a reduced global DNA methylation

**Figure 1:**
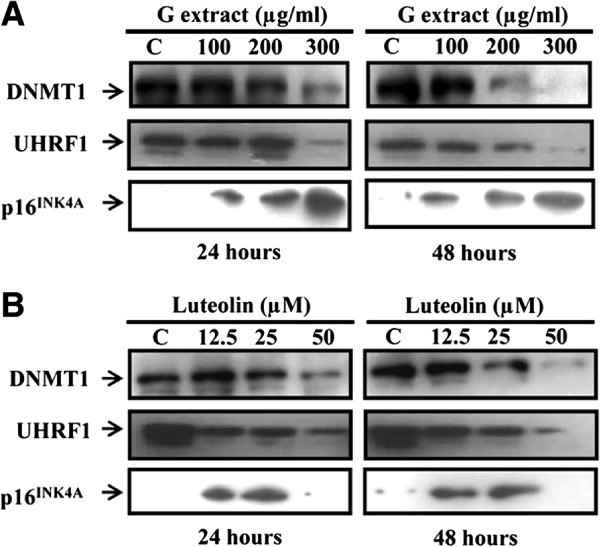
**Aqueous gall extract and luteolin induce UHRF1 and DNMT1 down-regulation and p16**^**INK4A **^**up-regulation in HeLa cells.** HeLa cells were exposed to G extract (**A**) or luteolin (**B**) at the indicated concentrations for 24 and 48 hours. DNMT1, UHRF1 p16^INK4A^ were analyzed by western blotting. Results were representative of three separated experiments.

The present study was undertaken to investigate the effect of G extract on the expression of UHRF1/DNMT1 tandem known to be involved in gene expression regulation via DNA methylation [9,11]. HeLa cells were treated with different concentrations (100, 200 and 300 μg/ml) of G extract for 24 and 48 hours. As shown in Figure 1A, treating the cells with 300 μg/ml of G extract for 24 hours induced a significant decrease in the expression of UHRF1, DNMT1 and this expression was abolished after 48 hours of treatment. Cells treatment with 200 μg/ml of G extract also induced a significant decrease of UHRF1 and DNMT1 expressions but only after exposure for 48 hours whereas at 100 μg/ml there was no effect. Several studies have been shown that UHRF1 negatively regulates the expression of the *p16*^*INK4A*^ tumor suppressor gene [19,36]. Thus, we aimed to know whether G extract and luteolin could affect the expression of p16^INK4A^ in HeLa cell line. Our results showed that G extract induced a dose dependently up-regulation of p16^INK4A^ expression (Figure 1A). This effect was associated with the G extract-induced down-regulation of UHRF1 and DNMT1 expression (Figure 1A). Quantitative phytochemical analysis of G extract showed that flavonoids are the major compounds present in this extract, which suggest that G extract-induced effect on UHRF1 and DNMT1 expression could be attributed, at least in part to these compounds. In order to obtain evidence for this hypothesis, the effect of luteolin, a dietary flavonoid on the expression of UHRF1, DNMT1 and p16^INK4A^ proteins has been investigated. As shown in Figure 1B, treating cells with luteolin induced a dose and time down-regulation of UHRF1. Indeed, UHRF1 expression was significantly decreased after 24 hours treatments and approximately disappeared at 50 μM after 48 hours (Figure 1B). For DNMT1, only 50 μM induced a significant decrease of DNMT1 expressions after incubation for 24 hours. After treatment of cells for 48 hours, DNMT1 expression was significantly decreased at 25 μM and totally abolished at 50 μM whereas at 12.5 μM there was no effect (Figure 1B).

Next, we wanted to determine whether the reduced UHRF1 and DNMT1 levels in G extract and luteolin-treated HeLa cells are correlated with an effect on genomic DNA methylation. We assessed global genomic DNA methylation by Imprint® Methylated DNA Quantification assay. As shown in Table 2, a general decrease in genomic DNA methylation was evidenced by both natural products. Indeed, our results demonstrate that G extract and luteolin inhibited DNA methylation as compared to untreated cells (Table 2) with percent inhibition of 42.4% ± 1.6% and 46.5% ± 1.1% in the presence of G extract and luteolin, respectively. Altogether, these findings showed that both G extract and luteolin were able to decrease UHRF1 and DNMT1 expression leading to a reduced genomic DNA methylation which could induce the re-expression of the *p16*^*INK4A*^ tumor suppressor gene.

**Table 2:** Effects of aqeous gall extract and luteolin on global methylated DNA in HeLa cells

**Average of absorbance (nm)**		**Methylated DNA (% of control)**
**MC**	**0.662 ± 0.030**	259.90* ± 4.9
**C**	**0.283 ± 0.001**	**100.00**
**G200**	**0.152 ± 0.003**	53.53* ± 1.52
**L25**	**0.163 ± 0.005**	57.60^* ^**± 2.29**

Total DNA was isolated from HeLa cancer cells using QIAamp^®^ DNA Kit. the content of methylated DNA was determined using 200 ng of DNA from untreated cells (C), treated cells with 200 μg/ml of G extract (G200 or with 25 μM of luteolin(L25) for 48 hours and the commercial methylated control (MC) (Imprint Methylated DNA Quantification Kit) Values are means ± *S.E.M*. of three independent experiments. Statistically significant, *P < 0.001 (versus the untreated cells).

### G extract and luteolin inhibit cell growth and induce cell cycle arrest of HeLa cells

**Figure 2:**
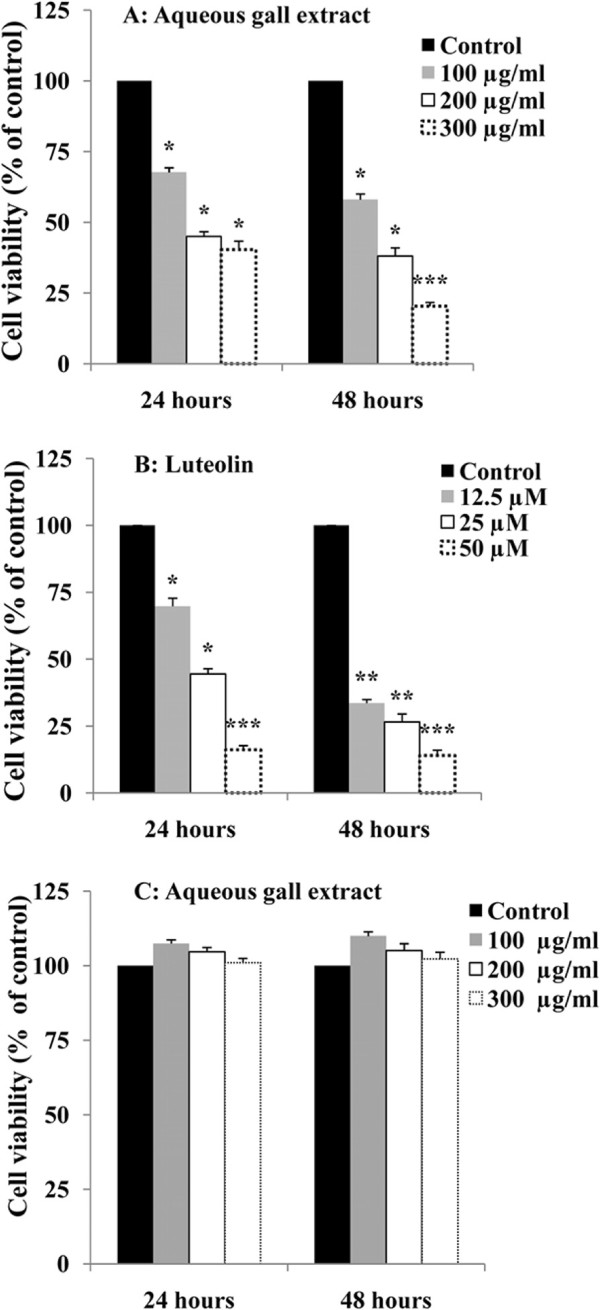
**Aqueous gall extract and luteolin inhibit HeLa cell proliferation.** HeLa cells and primary cultured human foreskin keratinocytes were treated with different concentrations of G extract (**A** and **C**) or luteolin (**B**) for 24 and 48 hours. Cell proliferation rate was assessed by colorimetric measurements using the MTT assay. The absolute value obtained for each G extract- or luteolin -treated sample is expressed in a second step as percent relative to the corresponding absolute value obtained for the untreated sample and set at 100. Values are means±S.E.M. of three independent experiments. Statistically significant, *P < 0.05, **P < 0.01, ***P < 0.001 (versus the corresponding untreated group).

Considering that *p16*^*INK4A*^ tumor suppressor gene is a downstream target of UHRF1 and a negative regulator of cell proliferation [17,36], we then wanted to determine whether G extract- or luteolin-induced up-regulation of p16^INK4A^ leads to cell proliferation inhibition and cell cycle arrest. As illustrated in Figure 2, exposure of HeLa cells to G extract (A) or luteolin (B) inhibited cell proliferation in a dose- and time-dependent manner. The IC_50_ values were determined graphically and the inhibition percentages were calculated. Inhibition of proliferation of HeLa cells, by G extract, reached a maximum of 79.6% and 59.7% at a concentration of 300 μg/ml after 48 and 24 hours of incubation, respectively (Figure 2A). IC_50_ values were 170 μg/ml and 140 μg/ml of G extract after 24 and 48 hours treatment, respectively. Interestingly, G extract had no effect on normal human keratinocytes when cells were treated with similar concentrations for 24 and 48 hours (Figure 2C). This suggests that G extract specifically targets cancer cells.

Luteolin was also able to induce cytotoxicity in HeLa cells (Figure 2B) with an IC_50_ value of 21.8 μM after 24 hours. At 50 μM, luteolin decreased proliferation of HeLa cells by 83.8% and 85.9% after 24 hours and 48 hours of incubation, respectively. These results indicate that both natural products induce a dose-dependent cell growth inhibition of HeLa cells.

**Figure 3:**
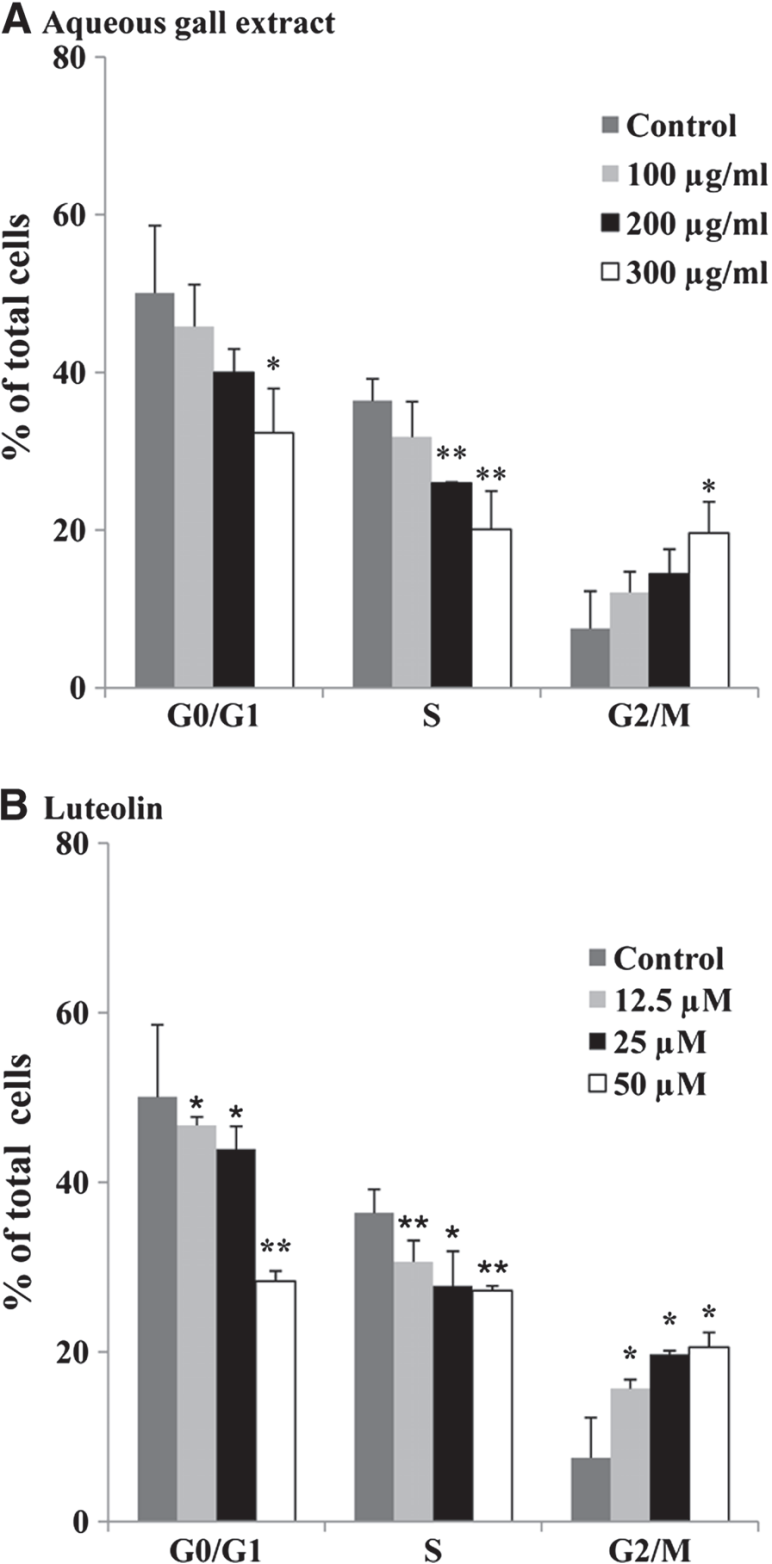
**Aqueous gall extract and luteolin arrest cell cycle progression.** Cells were treated with different concentrations of aqueous gall extract (**A**) or luteolin (**B**) for 24 hours. Cell cycle distribution was assessed by a capillary cytometry detection assay. Cell number in G0/G1, S or G2/M phase was determined and expressed as percent relative to the total cell number. Values are means ± S.E.M. of three experiments. Statistically significant, *P < 0.05, **P < 0.01, (versus the corresponding untreated group).

Because cell proliferation is a consequence of the progression of the cells through the different phases of the cell cycle, we next determined the effects of G extract and luteolin on the cell cycle distribution (Figure 3). HeLa cells were incubated in the presence and/or absence of different concentrations of G extract (A) or luteolin (B) for 24 hours. Treatment of HeLa cells with the extract caused an increase in G2/M peaks and a decrease in the S and G0/G1-phases fraction in a concentration-dependent manner (Figure 3A). Indeed, the percentage of cells in the G0/G1 phase was decreased from 50.1% (control) to 32.3% at 300 μg/ml whereas an accumulation of the cell population was observed in the the G2/M from 7.5% in untreated cells to 19.6% at the same concentration. Similarly to G extract, treatment of HeLa cells with luteolin caused an increase in G2/M phase and a decrease in the G0/G1-phase fraction in a concentration-dependent manner (Figure 3B). It appears therefore that G extract is able to inhibit the proliferation of Hela cells by promoting cell cycle arrest at the G2/M phase.

### G extract and luteolin induce apoptosis in HeLa cells

**Figure 4:**
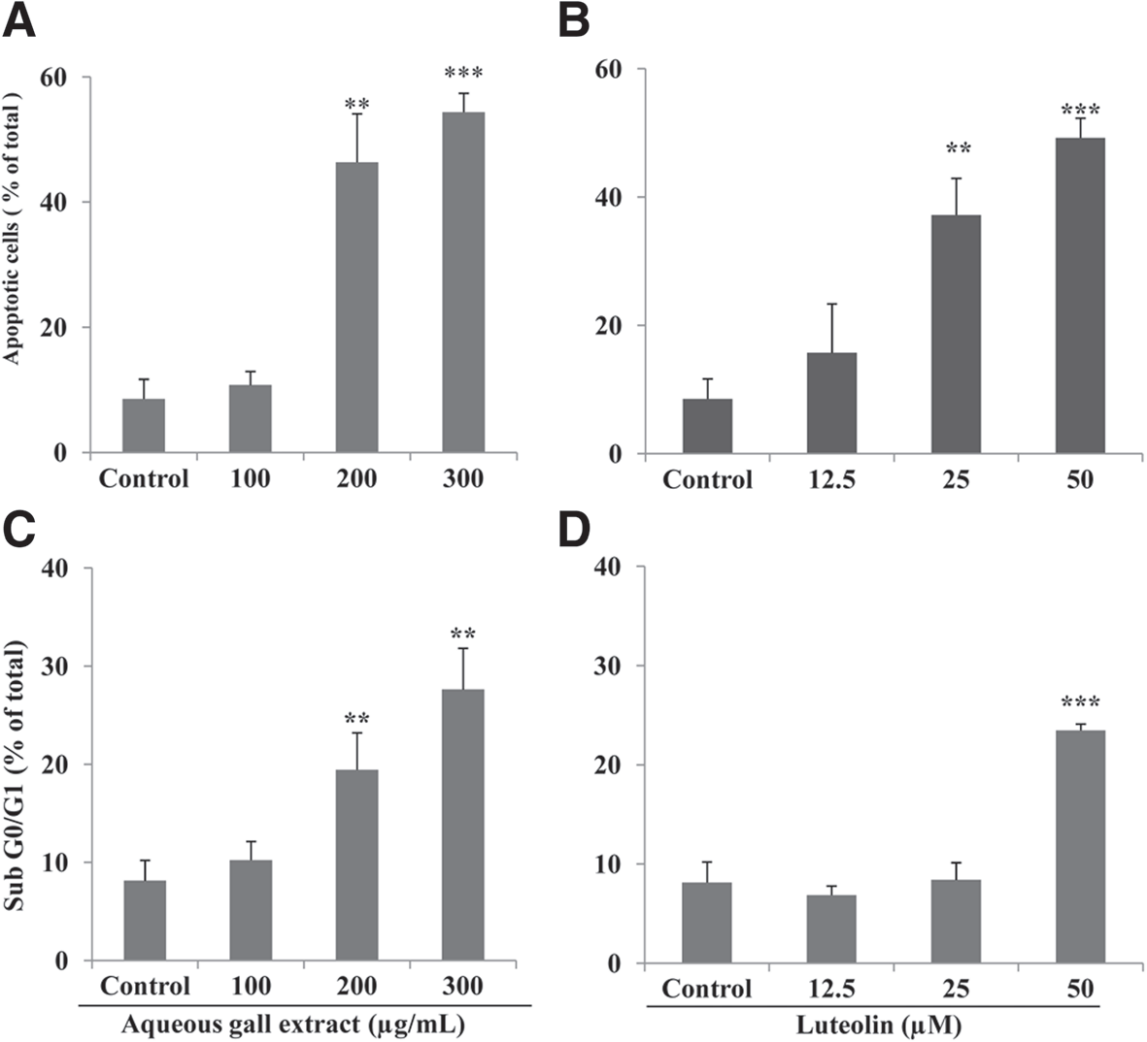
**Aqueous gall extract and luteolin induce HeLa apoptosis.** Cells were treated with different concentrations of aqueous gall extract (**A**, **C**) or luteolin (**B**, **D**) for 24 hours. Cell apoptosis rate was assessed by capillary cytometry using the Annexin V-FITC staining assay. The number of apoptotic cells is expressed as percent relative to the total cell number. Cell number in subG0/G1, phase was determined and expressed as percent relative to the total cell number. Values are means ± S.E.M. of three experiments. Statistically significant, *P < 0.05, **P < 0.01, ***P < 0.001 (versus the corresponding untreated group).

UHRF1 down-regulation has been shown to induce apoptosis in cancer cells [37]. Moreover, it has recently been demonstrated that UHRF1 down-regulation inhibits cell growth and induces apoptosis of colorectal cancer through p16^INK4A^ up-regulation [17]. Thus, we next investigated whether G extract- or luteolin-induced UHRF1 down-regulation and p16^INK4A^ up-regulation could induce apoptosis in HeLa cells. As shown in Figure 4, increasing concentrations of both products are associated with increasing number of apoptotic cells. Indeed, the percentage of apoptotic cells significantly increased from 8.6 (control) to 46.4% at 200 μg/ml and 54.4% at 300 μg/ml of G extract (Figure 4A), and to 37.2% at 25 μM and 49.2% at 50 μM of luteolin (Figure 4B). As expected, the calculated half-maximal effect of G extract on apoptosis was 170 μg/ml for 24 hours of treatment. Hence, these data were consistent with those obtained from cell proliferation assays (Figure 2). As a next step, cell cycle phase distribution analysis was focused on the detection of specific G0/G1 apoptotic cells; as shown in Figure 4, increasing concentrations of G extract led to increasing number of hypodiploid sub-G0/G1 cells (Figure 4C). Thus, G extract induced an increase in sub-G1 peak in a concentration-dependent manner ranged from 10.2% to 27.6% at concentrations of 100 and 300 μg/ml, respectively. At 50 μM of luteolin, an increment from 8.1% (control) 23.5% was observed in subG1 phase (Figure 4D). Finally, all these results suggest the occurrence of apoptosis in HeLa cells related to UHRF1 down-regulation and p16^INK4A^ up-regulation when exposed to G extract or luteolin.

## Discussion

Several studies have reported that plant-derived natural products have cancer chemopreventive and chemotherapeutic properties. Polyphenol-rich fruits and vegetables have been suggested to have anti-cancer properties in several cancers [3,38]. The aim of the present study was to determine the anti-proliferative and pro-apoptotic potential of G extract, a source rich in polyphenols (63%, data not shown) on the human cervical cancer HeLa cell line and if so, to characterize the mechanism involved. The present study indicates that G extract markedly inhibited proliferation of human cervical cancer HeLa cell line in a concentration-dependent manner. The G extract-induced growth inhibitory effect is associated with an arrest of the cell cycle progression in G2/M phase as shown by the cell phase distribution. In addition, G extract promoted in a concentration-dependent manner these cells towards apoptosis as indicated by annexin V labelling and by the increase in hypodiploid sub-G0/G1 cell population. In order to characterize the mechanism involved in the anti-proliferative and pro-apoptotic signalling pathway activated by G extract, the expression of the anti-apoptotic UHRF1, its main partner DNMT1 and the cell cycle inhibitor p16^INK4A^ was determined. The present findings indicate that G extract caused a marked and concentration-dependent up-regulation of p16^INK4A^ suggesting that this cell cycle checkpoint regulator is a key mediator of the pro-apoptotic activity of G extract in HeLa cells. In addition, G extract also caused a parallel down-regulation of the anti-apoptotic UHRF1 and its partner DNMT1. Similarly, the natural anti-cancer drug, epigallocatechin-3-gallate has been shown to induce p16^INK4A^ re-expression-dependent pro-apoptotic pathway via the down-regulation of UHRF1 in Jurkat cells [19]. Moreover, a recently published study has shown that UHRF1 depletion in cancer cells causes G2/M cell cycle arrest and apoptosis accompanied with phosphorylation of cyclin-dependent kinase 1 (CDK1) [37] which is in agreement with our present data. UHRF1 is an oncogene protein known to bind to methylated DNA and to recruit the DNMT1 to regulate tumor suppressor gene expression including p16^INK4A^[38]. Here, we showed that G extract decreased the expression UHRF1 as well as DNMT1. This effect was accompanied with an up-regulation of tumor suppressor gene *p16*^*INK4A*^. As UHRF1 is a negative regulator of p16^INK4A^ expression involving DNMT1 [19,36], our results suggest that the mechanism of action of G extract involves, at least in part, a down-regulation of UHRF1 with subsequent down-regulation of DNMT1 leading to an up-regulation of *p16*^*INK4A*^ gene inducing G2/M cell cycle arrest. In agreement with this hypothesis, we have recently shown that curcumin inhibited melanoma cell proliferation and cell cycle progression by accumulating cells at the G2/M-phase with decreased expression of UHRF1 and DNMT1 and enhanced expression of p21, a p16^INK4A^ -homolog [39]. Furthermore, because of CDK1 is required for progression of cells from the G2 phase into and through mitosis, down regulation of UHRF1 after cell treatment with G extract might also induce CDK1 phosphorylation and causes the G2/M cell cycle arrest and apoptosis as previously described in UHRF1 depleted cells [37].

Considering that G extract has a high quantity of polyphenolic compounds, we hypothesized that these products could be involved in the anti-proliferative and pro-apoptotic effects on HeLa cells. So, in order to obtain evidence for this hypothesis, the dietary flavonoid luteolin has been used in this study. Several studies have shown that flavonoids have anti-cancer effect on cancer cells involving several mechanisms including, cancer cells elimination, cell-cycle progression inhibition and induction of apoptosis [40-42]. Our results indicate that luteolin inhibits cell proliferation, arrests cell cycle progression and induces apoptosis in HeLa cells. A similar mechanism has also been involved in the effect of luteolin on cell cycle and apoptosis in HeLa cancer cells [43]. It has been shown that luteolin is able to induce G2/M cell cycle arrest and apoptosis in human oesophageal squamous cell carcinoma cell line (KYSE-510) cells in a dose- and time-dependent manner through p73-dependent up-regulation of p21^waf1^ and down-regulation of cyclin B1 [44]. In the present study, we found that luteolin induced cell cycle arrest and apoptosis in HeLa cells associated with a decrease in the expression of UHRF1 and DNMT1 and an increase in the expression of *p16*^*INK4A*^. As p73 is a negative regulator of UHRF1 [45] and a positive regulator of p16^INK4A^[46], luteolin-induced UHRF1/ p16^INK4A^ deregulation observed in HeLa cells could be a result of p73 up-regulation. Similarly, Aronia melanocarpa juice, rich resource in polyphenols has been shown to induce p73-dependent pro-apoptotic pathway involving UHRF1 down-regulation in the p53- deficient acute lymphoblastic leukemia Jurkat cell line [3]. UHRF1 plays an important role in cancer progression through epigenetic mechanisms. However, several reports indicated that UHRF1 contributes to silencing of tumor suppressor genes by recruiting DNMT1 to their promoters. Conversely, demethylation of tumor suppressor gene promoters has been ascribed to some anti-cancer natural products such as epigallocatechin-3-O-gallate [47,48]. Our data showed that both luteolin and G extract were able to down regulate UHRF1 and DNMT1 expressions in HeLa cells. This effect was associated with re-expression of tumor suppressor gene p16^INK4A^. Unexpectedly, p16^INK4A^ was totally repressed at the higher concentration (50 μM) of luteolin which could result from p16^INK4A^ protein denaturation and/or degradation at this concentration. In agreement with this suggestion, luteolin has been shown to up-regulate p21 expression at low concentrations and to down-regulate its expression at high concentrations [49].

Emerging evidence suggests that dietary natural products are involved in epigenetic modifications, especially DNA methylation leading to reduce the risk of cancer [50,51]. Here, we examined the effect of G extract and luteolin on the global DNA methylation in HeLa cells. Our results reveal that the levels of global DNA methylation were reduced in HeLa cells by about 42.4% and 46.5% in the presence of G extract and luteolin for two days, respectively. This effect was associated with a sharp decrease in the expression of DNMT1. The inhibition of DNA methylation as well as UHRF1 and DNMT1 down-regulation and the re-expression of p16^INK4A^ may be ascribed to several compounds found in G extract. Preliminary results of phytochemical screening revealed the presence of polyphenols. Furthermore, it was reported that *L. guyonianum* ethyl acetate extract contains epigallocatechin-3-O-gallate [52]. This biologically active substance could induce p16^INK4A^ re-expression through UHRF1 and DNMT1 depletion [19]. Our data support the idea that the DNA methylation process can be reversed in cancer cells by bioactive phytochemicals. Indeed, it has been shown that apple polyphenols has potent DNA demethylation activity in colorectal cancers by reducing DNMT1 expression with a subsequent activation of TSGs such as *p16*^*INK4A*^[53]. In the same way, it has been shown that grape seed proanthocyanidins, an anti-carcinogenic product, caused a reduced global DNA methylation in skin cancer cells related to a decrease in the level of DNMT1 and an increase in the level of p16^*INK4A*^[18]. Considering that UHRF1 binds to methylated promoter of p16^INK4A^[54] and that UHRF1 interacts with DNMT1 and regulates its expression [36], it is likely that G extract and luteolin induce in cervical cancer cells a down-regulation of UHRF1 with subsequent decrease of DNMT1 expression causing demethylation of p16^INK4A^ promoter.

## Conclusion

This is the first report which shows that G extract induces apoptosis related to a reduced DNA methylation likely by acting on the epigenetic integrator UHRF1 and its main partner DNMT1. By using cervical cancer HeLa cell line, we have shown that G extract inhibits cell proliferation and arrests cell cycle progression at the G2/M phase which could be through re-expression the tumor suppressor gene *p16*^*INK4A*^.

## Abbreviations

G extract: Aqueous gall extract; DNMT1: DNA methyltransferase1; TSGs: Tumor suppressor genes; UHRF1: Ubiquitin-like, containing PHD and RING finger domains, 1.

## Competing interests

The authors declare that they have no competing interests.

## Authors’ contributions

MK, MA, CB and MM designed the experiments and the draft. CDM, JPG, LCG, KG and YM equally contributed to the writing the article. All authors read and approved the final manuscript.
